# Epigenetic Regulation of the Ontogenic Expression of the Dopamine Transporter

**DOI:** 10.3389/fgene.2019.01099

**Published:** 2019-11-04

**Authors:** Ashley L. Green, Aseel Eid, Le Zhan, Helmut Zarbl, Grace L. Guo, Jason R. Richardson

**Affiliations:** ^1^Environmental and Occupational Health Sciences Institute and Department of Environmental and Occupational Medicine, Rutgers Robert Wood Johnson Medical School, Piscataway, NJ, United States; ^2^Department of Environmental Health Sciences, Robert Stempel School of Public Health and Social Work, Florida International University, Miami, FL, United States; ^3^Department of Pharmacology and Toxicology, Ernest Mario School of Pharmacy, Rutgers University, Piscataway, NJ, United States

**Keywords:** dopamine transporter, ontogeny, epigenetics, histone modifications, *Nurr1*, *Pitx3*

## Abstract

The dopamine transporter (DAT) is a plasma membrane transport protein responsible for regulating the duration and intensity of dopaminergic signaling. Altered expression of DAT is linked to neurodevelopmental disorders, including attention deficit hyperactivity disorder and autism spectrum disorder, and is shown to contribute to the response of psychotropic drugs and neurotoxicants. Although the postnatal levels of DAT have been characterized, there are few data regarding the mechanisms that regulate postnatal DAT expression. Here, we examine the ontogeny of DAT mRNA from postnatal days 0 to 182 in the rat brain and define a role for epigenetic mechanisms regulating DAT expression. DAT mRNA and protein significantly increased between PND 0 and 6 months in rat midbrain and striatum, respectively. The epigenetic modifiers *Dnmt1*, *Dnmt3a*, *Dnmt3b*, and *Hdac2* demonstrated age associated decreases in mRNA expression whereas *Hdac5* and *Hdac8* showed increased mRNA expression with age. Chromatin immunoprecipitation studies revealed increased protein enrichment of acetylated histone 3 at lysines 9 and 14 and the dopaminergic transcription factors Nurr1 and Pitx3 within the DAT promoter in an age-related manner. Together these studies provide evidence for the role of epigenetic modifications in the regulation of DAT during development. The identification of these mechanisms may contribute to potential therapeutic interventions aimed at neurodevelopmental disorders of the dopaminergic system.

## Introduction

The dopamine transporter (DAT) is a twelve transmembrane transporter which is recognized as the primary modulator of dopaminergic signaling within the brain (Vaughan and Foster, 2013). The functional roles of DAT within the dopaminergic system are well characterized *in vitro* and in mature mammalian models and include the regulation and maintenance of dopamine levels (Giros and Caron, 1993; Nirenberg et al., 1996; Chen and Reith, 2000; Falkenburger et al., 2001; Ingram et al., 2002; Gorentla and Vaughan, 2005). These actions facilitate central nervous system processes such as attention, learning, cognition, motor activity, and mood. Consequently, alterations in dopaminergic transmission and DAT expression stand to adversely affect these neurological activities resulting in behavioral disorders (Bannon et al., 2001; Lin et al., 2011). Dysregulated DAT is associated with neurodevelopmental disorders such as autism spectrum disorder (ASD) and attention deficit hyperactivity disorder (ADHD) (Volkow et al., 2007; Nakamura et al., 2010; Makkonen et al., 2011; Spencer et al., 2013). In addition to controlling dopamine levels within the synapse, DAT is the target of both therapeutic and illicit compounds (Nutt et al., 2004; Runyon and Carroll, 2006; Zhu and Reith, 2008; Schmitt and Reith, 2010; Dela Pena et al., 2015). These compounds carry out their neurobehavioral effects by enhancing, antagonizing or altering DAT expression, which subsequently changes DA signaling (Goodwin et al., 2009; Siciliano et al., 2016). The significant role of DAT in controlling dopaminergic transmission provides the incentive for studies aimed at understanding its regulation.

Although originally thought to have a relatively long half-life (Rego et al., 1999), DAT has since demonstrated to have a half-life of about 2 days suggesting a more dynamic process of transcriptional and translational regulation (Kimmel et al., 2000; Kahlig and Galli, 2003). Several studies have characterized the developmental expression of DAT *in vivo* yet none have explored the mechanisms responsible for the changes in expression levels. Following many cloning and localization studies of DAT (Giros et al., 1991; Kilty et al., 1991; Shimada et al., 1991; Usdin et al., 1991), the developmental mRNA expression profiles of the gene were characterized. Using autoradiography and *in situ* hybridization techniques, these studies showed DAT mRNA expression is first detected in early embryonic days and expression levels peak within the first month after birth in rodents (Lauder and Bloom, 1974; Fujita et al., 1993; Tison et al., 1994; Coulter et al., 1996; Coulter et al., 1997; Tarazi et al., 1998; Moll et al., 2000; Galineau et al., 2004). Despite this, the mechanisms involved in DAT developmental regulation remain unclear.

Previously, our lab and others have employed *in vitro* methods to evaluate the molecular mechanisms involved in DAT gene regulation (He et al., 2011; Green et al., 2015; Green et al., 2017). The dopaminergic transcription factors Nurr1 and Pitx3 are essential for the development, survival and maintenance of midbrain dopaminergic neurons (Lee et al., 2010; Rodriguez-Traver et al., 2016; Salemi et al., 2016). These transcription factors also demonstrate cooperative binding to the DAT promoter to enhance gene expression (Martinat et al., 2006). In conjunction with other studies, we and others have shown that histone acetylation and DNA methylation are involved in altered DAT mRNA expression *in vitro* (Wang et al., 2007; Shumay et al., 2010; He et al., 2011; Green et al., 2015; Green et al., 2017), suggesting the potential for epigenetic mechanisms in DAT gene regulation *in vivo*. Although *in vitro* evidence firmly suggests the role of transcription factors and epigenetic mechanisms in DAT regulation, no studies have explored their contributions during *in vivo* development. In this study we assess DAT mRNA expression during postnatal stages of rat brain development and evaluate the relative contributions of epigenetic modifications and transcription factors to changes in gene expression.

## Methods

### Animals

Ten-week-old Long-Evans rat breeding pairs were purchased from Charles River Laboratories (Wilmington, MA). Rats were housed according to the American Animal Association Laboratory Animal Care guidelines and bred under standard conditions at Rutgers University School of Public Health. Rats were maintained on a 12:12 light/dark cycle, and food and water were available *ad libitum*. Brains from male offspring were collected at postnatal day (PND) ages: 0, 3, 7, 14, 28, 56, and 182 (approximately 6 months). Midbrain and striatum were immediately frozen in liquid nitrogen and stored at −80°C. The International Animal Care and Use Committee at Rutgers University approved all animal handling procedures.

### RNA Isolation and cDNA Synthesis

Total RNA was isolated from midbrain tissue using the Qiagen RNeasy Kit (Qiagen, Hilden, Germany) as described previously (Hossain and Richardson, 2011). RNA concentration was determined using the NanoDrop 2000 spectrophotometer (NanoDrop Technologies, Wilmington, DE) at 260 nm wavelength. One µg of RNA was used for cDNA synthesis with the First Strand Synthesis Kit (Invitrogen, Carlsbad, CA) following the manufacturer instructions.

### Real-Time Quantitative Polymerase Chain Reaction

Rat primer sequences (Table 1) were obtained from PubMed. Single amplicon primers were designed using the National Center for Biotechnology Information PrimerBLAST application (http://www.ncbi.nlm.nih.gov/tools/primer-blast/index.cgi). A single PCR product of expected amplicon size was confirmed by agarose gel electrophoresis (data not shown). Reactions were prepared in a total volume of 25 µl using SYBR Green Master Mix (Applied Biosystems, Foster City, CA). qPCR was performed using the ViiA7^™^ Real-Time PCR system using the following conditions: 2 min at 50°C and 10 min at 95°C, followed by 40 cycles of 95°C for 15 s and 1 min at the annealing temperature for each primer set. All samples were run in duplicate and analyzed using the 2^−ΔΔCT^ (Livak and Schmittgen, 2001) with TATA binding protein used as a normalizer for each gene.

**Table 1:** Primers used for qPCR ChIP, and pyrosequencing methods. qPCR gene target sequences were obtained from the mRNA nucleotide sequence available on PubMed. Primers were generated in PrimerBLAST each with a product length of 150–200 bp and all span exon-exon junctions. ChIP target gene primers were obtained from a previously published manuscript (He et al., 2011). Each of the ChIP target primer pairs encompasses 200 bp regions of the DAT promoter. The pyrosequencing primers were purchased from Qiagen.

Gene Target	Primer Sequence (5’→3’)
rDAT	F: GAGGTTTCCCTACCTGTGCT
	R: GTGAAGCCCACACCTTTCAG
rDNMT1	F: GCCCCATGAAACGCTCTAAG
	R: GTGGGTGTTCTCAGGCCTAT
rDNMT3a	F: AAGGCACTCGCTGGGTCAT
	R: AGGACTTCGTAGATGGCTTTGC
rDNMT3b	F: AAATCCAGGGACTTGCAGGAA
	R: GGTCTCTGGTGTACAGACTGG
rHDAC1	F: CAGAAGCCAAAGGGGTCAAA
	R: AAAATCTGAGAAATTGAGGGAAAGT
rHDAC2	F: CTGCAGTTGCCCTTGATTGT
	R: CAGGCGCATGTGGTAACATT
rHDAC3	F: ACTTCGAGTACTTTGCCCCA
	R: GCCTCGTCAGTCCTGTCATA
rHDAC5	F: ATGGCCTTGGATGGGCATTA
	R: CGACTCGTTGGGAGAGTTCA
rHDAC8	F: ATACTTGACCGGGGTCATCC
	R: CCACATGCTTCAGATTCCCTTT
rTBP	F: CACCACCCCCTTGTATCCTT
	R: CAGCAAACCGCTTGGGATTA
**ChIP Target**	
DAT I	F: TACAGGACCTCAGAGCTGAA
	R: CCCTTAGTTCTGTGTGGAACG
DAT II	F: GCCTCTGGCTCCCCGCAGTC
	R: AGTCCCTCTTCACAGCTCTG
DAT III	F: AACCAGTCGTTGGGAGCCCA
	R: GGGCCGGCGAGGGGCTTGAC
DAT IV	F: CTTGCTTTGTCCCTGCGGAG
	R: GCGAGGTTGTCAGAAGCAGA
DAT V	F: ACCGCACTTGTGACCATAGG
	R: GGACGCACCGCCCGGTGCTG
DAT VI	F: TATAGGTTTCTCCAGGGAAA
	R: AATACGGATCCAGGGGTTGG
**Pyrosequencing Target**	**Primer Catalog Number**
DAT 1	PM00444052
DAT 2	PM00444059
DAT 3	PM00444066
DAT 4	PM00444073

### DNA Bisulfite Conversion and Pyrosequencing

DNA from frozen midbrain tissues of PND 3, 7 and 56 rats were isolated using DNeasy Blood and Tissue Kit (Qiagen) per manufacturer instructions. DNA concentrations were determined using the NanoDrop2000 spectrophotometer. One µg of DNA was used for bisulfite conversion using EpiTect Bisulfite Conversion Kit (Qiagen) according to the kit instructions. Bisulfite modified DNA was amplified using PyroMark PCR Kit per insert instructions. Briefly, 20 ng of bisulfite converted DNA was mixed with 1x of PyroMark PCR master mix, CoraLoad Concentrate and pre-designed CpG PCR Primer (Qiagen). PCR was performed using the following conditions: 95°C for 15 min, 45 cycles of 94°C for 30 s, 56°C for 30 s, 72°C for 30 s and a single elongation step at 72°C for 10 min. Single amplification products were verified by electrophoresis on a 1% agarose gel. A total of 15 µl of PCR product was used for pyrosequencing using the PyroMark Q24 System (Qiagen). The biotinylated PCR product was bound to streptavidin coated sepharose beads (GE Healthcare, Chicago, IL), washed, and denatured using the PyroMark vacuum prep tool. The single stranded PCR product was released into Advanced Annealing Buffer (Qiagen) containing 1× pre-designed CpG sequencing primer (Qiagen), heated at 80°C for 5 min and processed. The catalog number of primers used in these assays is located in Table 1. All assays included non-CpG cytosines to verify efficient bisulfite conversion and were performed in triplicate.

### Western Immunoblotting

Frozen striatal tissues were homogenized in buffer (320 mM sucrose, 5 mM HEPES, pH 7.4) containing protease inhibitor cocktail (Sigma-Aldrich) and centrifuged at 3,500 rpm for 5 min at 4°C. Supernatant was transferred to a fresh tube and spun at 14,000 rpm for 45 min at 4°C. Protein pellets were resuspended in buffer and supplemented with 0.1% protease inhibitor cocktail. Homogenate concentrations were determined using the Pierce^™^ bicinchoninic acid (BCA) assay kit (Thermo Scientific) and 20 µg of protein sample was loaded per lane on a 4–12% Bis-Tris Polyacrylamide Gel (Invitrogen). Membranes containing transferred proteins were blocked in milk solution (7.5% milk in 0.1% Tween 20 and Tris buffered saline) for 1 h. Membranes were incubated overnight at 4°C with DAT antibody (1:250; sc-14002 Santa Cruz) followed by 1 h room temperature incubation with anti-rabbit HRP conjugated secondary antibody. Membranes were visualized using Alpha Innotech Fluorochem Imaging System. The membrane was stripped using Pierce Stripping Buffer and re-probed with syntaxin to confirm equal loading.

### Chromatin Immunoprecipitation Assay

Frozen midbrain samples were crushed to a fine powder and crosslinked in fixation buffer (1× PBS pH 7.4, 1% formaldehyde and 0.1% protease inhibitor cocktail) for 10 min. The reaction was halted with 10× glycine to a final concentration of 0.125 M for 5 min. Tissue was pelleted at 2,000 rpm for 5 min and washed with cold PBS twice. The pellet was resuspended in cold cell lysis buffer (10 mM Tris pH 8, 10 mM NaCl, 3 mM MgCl_2_, 0.5% NP40) for 15 min. Nuclei were pelleted at 2,000 rpm for 5 min at 4°C and resuspended in nuclear lysis buffer (1% SDS, 5 mM EDTA, 50 mM Tris pH 8). Chromatin was prepared and quantitated as described previously (Green et al., 2017). Briefly, ChIP was performed with 4 µg of each antibody H3K9K14ac (cat # 9441, Cell Signaling), Nurr1 (cat # sc-991, Santa Cruz), Pitx3 (cat # 19307, Santa Cruz), Normal Rabbit IgG (cat# 12-370, Millipore) overnight at 4°C. Isolated DNA was subjected to qPCR to determine relative enrichment of histone and transcription factor proteins in DAT promoter regions. *Gapdh* levels were measured as a control using the same assay methods and antibodies. Primer sequences for qPCR were obtained from a previously published manuscript (He et al., 2011) and are found in Table 1. For each ChIP assay, data were normalized to input, IgG and represented as enrichment relative to control.

### Statistical Analyses

All data were analyzed and graphs generated using GraphPad Prism 7.0 Software (GraphPad Software, San Diego, CA). All experiments were performed in three or more replicates. Data are presented as mean ± SEM and analyzed by One-way analysis of variance (ANOVA), with the appropriate *post hoc* test indicated in the Results section and figure legends. Statistical significance was determined at level of p ≤ 0.05.

## Results

### Dopamine Transporter mRNA Expression and Protein Levels Peak at Postnatal day 56 in Developing Midbrain

**Figure 1:**
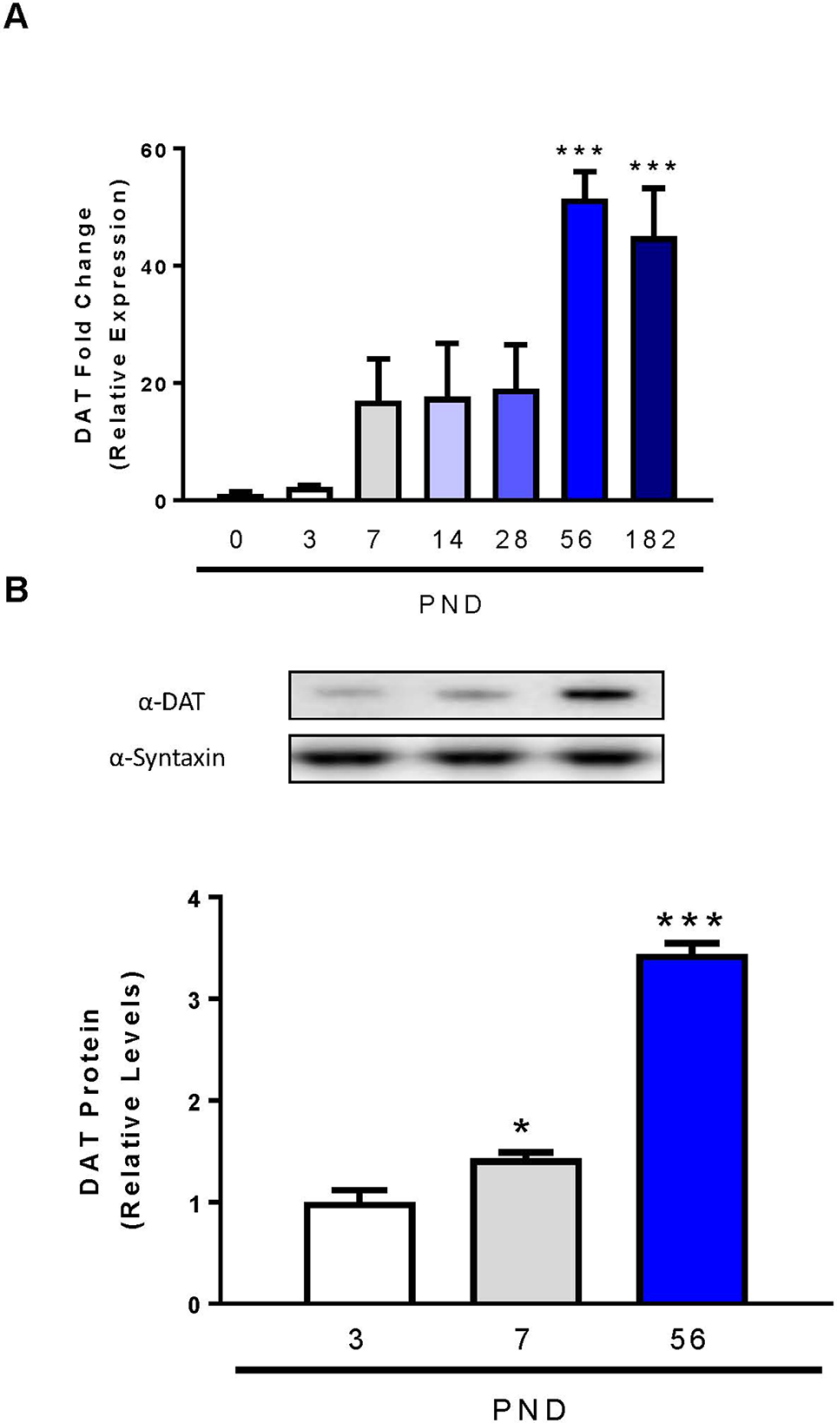
Dopamine transporter expression and levels in midbrain and striatum of developing rats. **(A)** DAT mRNA levels in the midbrain were analyzed with qPCR and normalized to TATA binding protein. **(B)** Quantification of DAT protein levels in the striatum were determined by western blot using syntaxin as loading control. Data represented as mean relative to PND 0 (mRNA) or PND 3 (protein) ± SEM; N = 3–4, *p ≤ 0.05 ***p ≤ 0.001. Data were analyzed by One-way ANOVA with Dunnett’s *post-hoc* with PND 0 as the control for mRNA and PND 3 for protein.

Previous studies used autoradiography or semi-quantitative *in situ* hybridization techniques to determine the initial expression, localization, and function of dopamine transporter mRNA and protein in the rat brain (Lauder and Bloom, 1974; Fujita et al., 1993; Tison et al., 1994; Galineau et al., 2004). In our study we utilized qPCR, as it provides more accurate quantitation and minimal amounts of RNA, and subsequently cDNA are required compared to *in situ* hybridization techniques (Hoebeeck et al., 2007). DAT mRNA is concentrated in the neurons of the ventral tegmental area and pars compacta of the substantia nigra, collectively identified as midbrain (Shimada et al., 1992; Cerruti et al., 1993). We performed qPCR on rat midbrain samples from PND 0 to 6 months of age to determine low, intermediate, and high expression periods for DAT postnatal mRNA expression (**Figure 1A**). Relative DAT mRNA increased two-fold between PND 0 and 3, followed by a 16-fold increase in DAT mRNA by PND 7 which persists until PND 28. DAT mRNA continues to increase until PND 56, when we detect maximum levels of a 51-fold increase and statistical significance has been reached (F_6,14_ = 9.552, p = 0.0003). Immunoblotting of developing rat striatum shows an age-dependent increase in DAT protein levels (**Figure 1B**). One-way ANOVA revealed a significant increase in protein levels of DAT within the striatum at PND 7 and PND 56 relative to PND 3 (F_2,6_ = 177, p < 0.0001). A 43% increase in DAT protein levels was measured at PND 7 (p = 0.0467), and a 344% increase at PND 56 (p < 0.0001). Collectively, these data demonstrate a peak at PND 56 for both mRNA and protein in the rat midbrain and striatum respectively.

### Transcriptional Profile of DNA Methylation Enzymes, Histone Deacetylases and Transcription Factors at Postnatal Days 3, 7, and 56 in rat Midbrain

**Figure 2:**
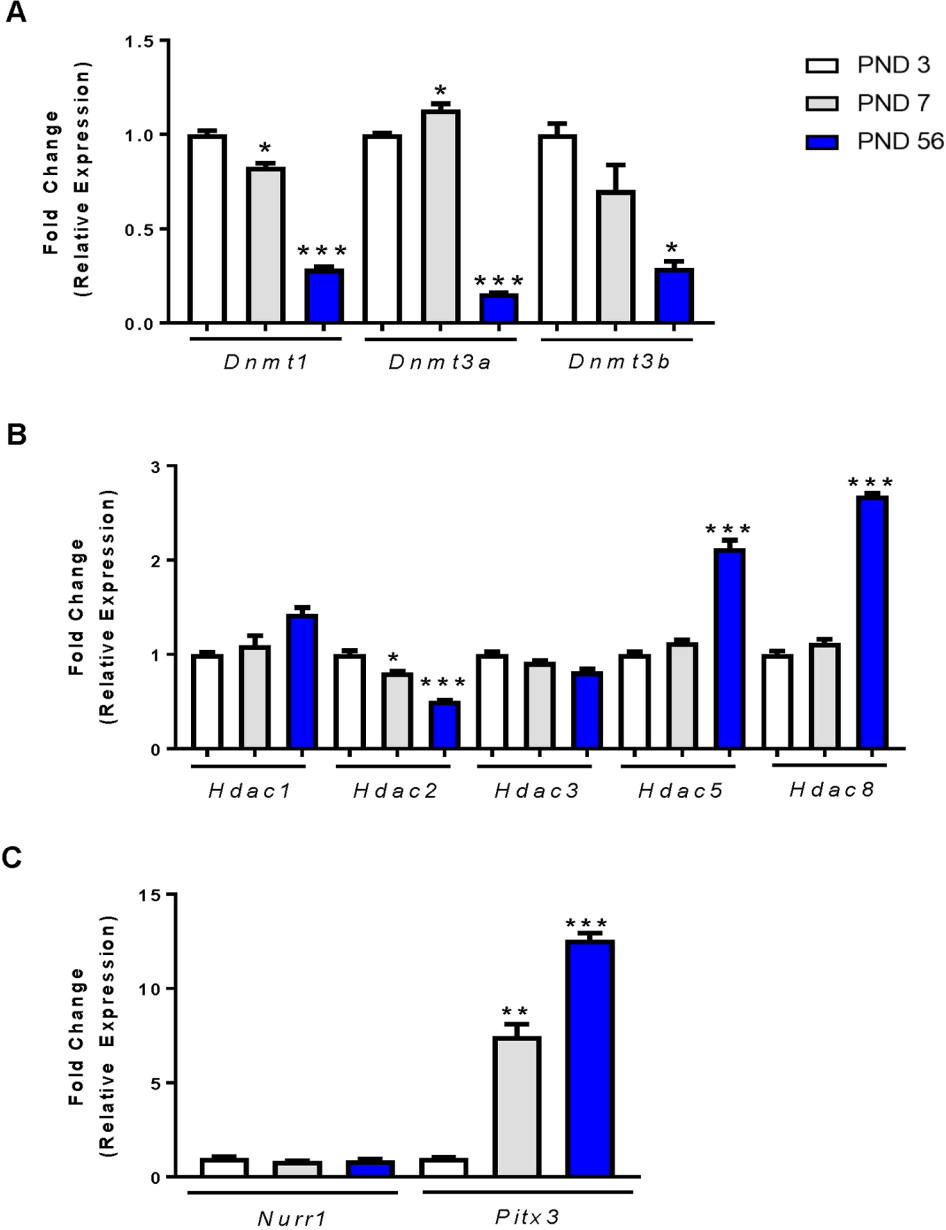
mRNA expression profiles of epigenetic regulators and transcription factors in the rat midbrain. **(A)** Fold change in DNMT expression relative to control (PND 3). **(B)** Fold change in HDAC expression relative to PND 3. **(C)** Fold change in Nurr1 and Pitx3 expression relative to PND 3. Data represented as fold change in expression ± SEM; N = 3–4, *p ≤ 0.05, **p ≤ 0.01,***p ≤ 0.001. Data were analyzed by One-way ANOVA with Dunnett’s *post-hoc* test holding PND 3 as the control.

Many studies have evaluated the gene and protein expression profiles of the DAT during development, yet few studies have focused on the mechanisms involved in these changes in expression. DNA methylation is an effective mechanism for silencing gene expression. The addition of methyl groups to the DNA is catalyzed by DNA methyltransferases (DNMTs). We evaluated mRNA expression of three DNMTs in the rat midbrain at PND 3, PND 7 and PND 56. For each DNMT enzyme, we report significant decreases in expression by one-way ANOVA, *Dnmt1* (F_2,9_ = 91.23, p < 0.0001), *Dnmt3a* (F_2,9_ = 214.6, p < 0.0001), and *Dnmt3b* (F_2,9_ = 6.915, p < 0.0001) (**Figure 2A**). *Dnmt1* expression decreased by 17.3% at PND 7 relative to PND 3 (p = 0.0222) and by 71.3% at PND 56 (p < 0.0001). *Dnmt3a* decreased by 84.3% at PND 56 (p < 0.0001), and *Dnmt3b* by 30% at PND 7 (not statistically significant) and by 71% at PND 56 (p = 0.0144).

Histone acetylation is another epigenetic modification characterized by the addition of acetyl groups to histone protein tails. These groups are added by histone acetyltransferases and removed by histone deacetylases (HDACs). We focused primarily on HDACs and their mRNA expression in the rat midbrain during PND 3, 7, and 56 (**Figure 2B**). The class I HDACs 1, 2, 3, and 8 expressed differently with age. There was no change in the expression of *Hdac1* and *Hdac3* at PND7, but a significant age-related decrease in *Hdac2* as measured by one-way ANOVA (F_2,9_ = 26.45, p = 0.0002). *Hdac2* mRNA was significantly decreased at PND 7 by 19.5% (p = 0.0342), and by 49% at PND 56 (p < 0.0001). Conversely, we measured a significant effect of age on *Hdac5* expression (F_2,9_ = 32.29, p < 0.0001), and *Hdac8* expression (F_2,9_ = 202.2, p < 0.0001). *Hdac5* expression was increased by 2.12-fold (p < 0.0001) and *Hdac8* expression was increased by 2.68-fold (p < 0.001) by PND 56.

The transcription factors Nurr1 and Pitx3 are important regulators of DAT expression (Sacchetti et al., 2001; Li et al., 2009) and shown by us and others to regulate DAT at the promoter region (Martinat et al., 2006; He et al., 2011; Green et al., 2017). We determined the relative mRNA expression profiles of each transcription factor in the midbrains of PND 3, 7, and 56 rats (**Figure 2C**). Analysis shows no change in Nurr1 gene expression during development. However, *Pitx3* mRNA was significantly increased (F_2,6_ = 94.18, p < 0.0001). We measured a 7.54-fold (p = 0.0012) increase in expression at PND 7 and an increase of 12.59-fold (p < 0.0001) by PND 56. These data display an age-related decrease in DNMTs, as well as an increase in *Hdac5*, *Hdac8* and the expression of the Pitx3 transcription factor. Together, these data may support or help explain the mechanism behind the increase in DAT mRNA and protein measured at PND 56.

### Pyrosequencing Reveals an Unmethylated DAT Promoter Across all Postnatal Days

**Figure 3:**
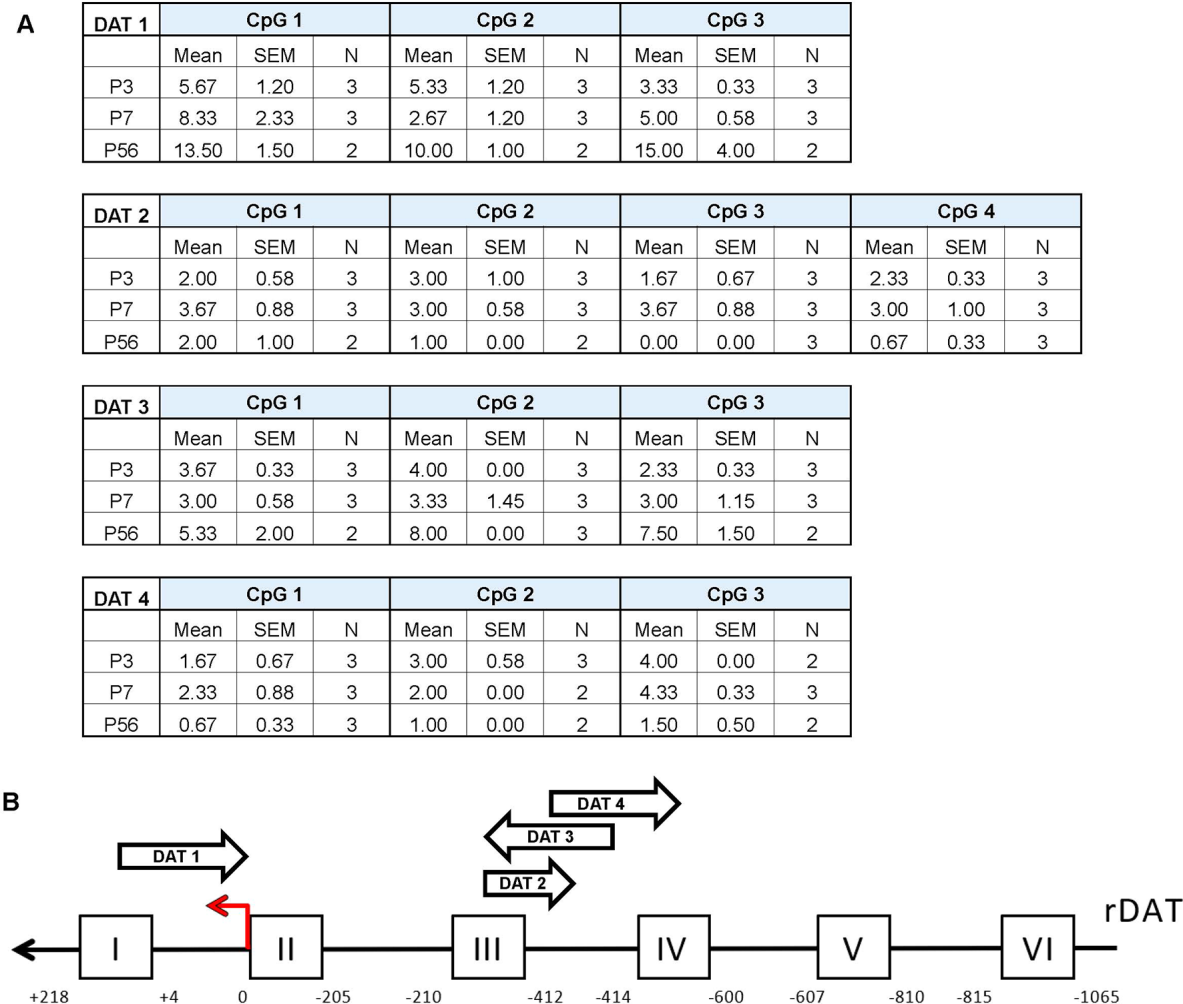
Pyrosequencing analysis of DNA methylation in developing rat midbrain. **(A)** Mean methylation level for each CpG site across the four DAT promoter regions examined. Data is represented as mean percent methylation, N = 2–3 and is indicated beside each data point. No statistical analyses were preformed on these data. **(B)** Diagram of the DAT promoter region analyzed in the study. The red arrow indicates the transcription start site (TSS position 0) while the position scale indicates how upstream (negative) or downstream (positive) the primers are in relationship to the TSS. The ChIP targets are presented in boxes with Roman numerals, and the approximate location of the pyrosequencing primers are indicated by arrows. Direction of these arrows indicates whether they target an area on the sense or antisense strand.

To further assess possible age-related changes in methylation patterns we performed pyrosequencing on the promoter of DAT (**Figure 3**). The epigenetic modification of DNA methylation involves the covalent addition of methyl groups to the C-5 position of cytosine residues at CpG dinucleotide sites (Moore et al., 2013) Following bisulfite conversion, we sequenced four promoter regions of the DAT and quantified the relative percent methylation at each CpG site. These data were then averaged across 2–3 rats per group to obtain a mean methylation level for each CpG site. Pyrosequencing results of the four promoter regions show a relatively unmethylated promoter which is consistent across all postnatal days. The approximate regions analyzed *via* pyromark assays are included in the scheme of the promoter in **Figure 3B**. No statistical analysis was performed on these data due to the low number of N’s quantified for these measurements.

### H3K9/K14 Acetylation Is Increased in Promoter Region 3 at PND 56

**Figure 4:**
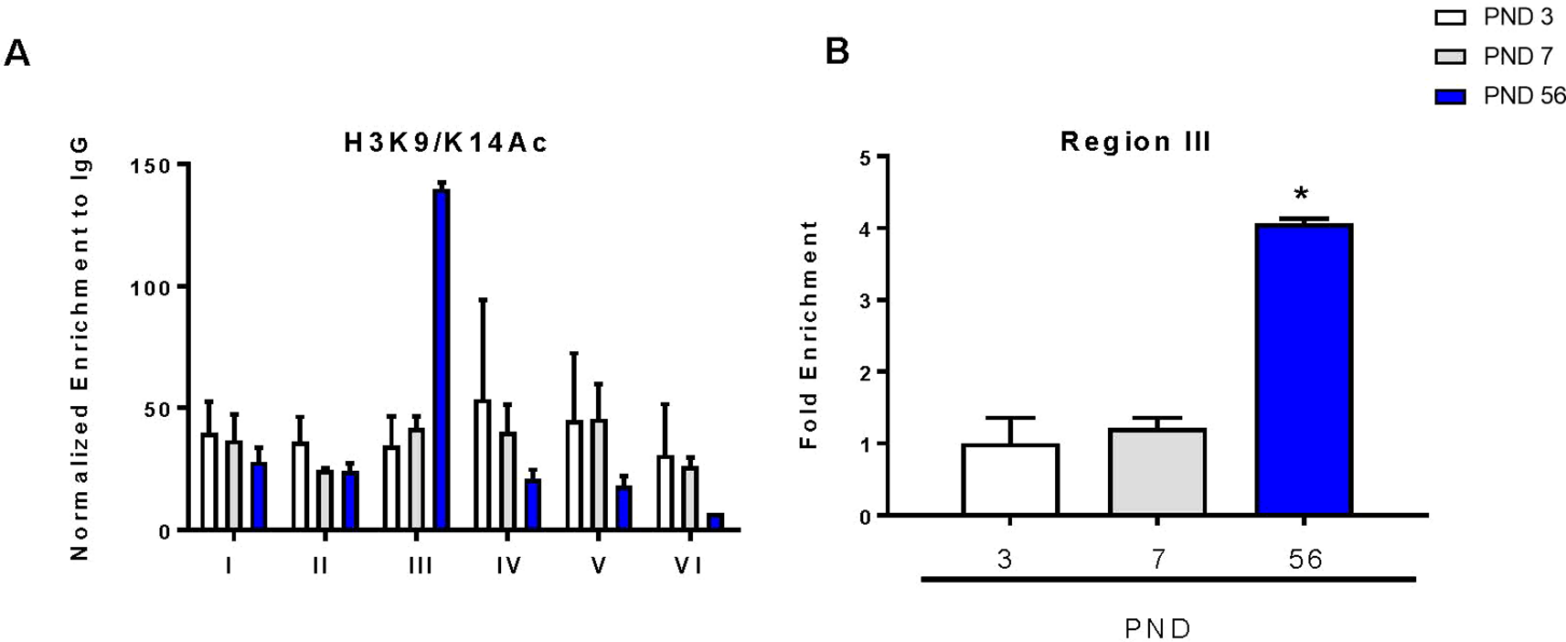
Chromatin immunoprecipitation of acetylated histone 3 on lysines 9 and 14 (H3K9/K14Ac) within the DAT promoter. **(A)** Enrichment of H3K9/K14Ac across six regions of the DAT promoter normalized to IgG. **(B)** Fold enrichment relative to PND 3 in the third region of the DAT promoter ± SEM; N = 3–4 *p ≤ 0.05. Data were analyzed by One-way ANOVA with Dunnett’s *post-hoc* relative to PND 3 for enrichment.

Acetylation within promoter regions is shown to enhance expression of genes. To determine the direct effects of histone acetylation on DAT gene expression during development, we immunoprecipitated acetylated histone at lysines 9 and 14 (H3K9/K14Ac) to evaluate its enrichment in the DAT promoter. We examined this by probing differences in six regions of the DAT promoter as previously described (He et al., 2011; Green et al., 2017) (**Figure 4A**). We measured a 100-fold enrichment (to IGG) in the PND 56 group compared to a 30-fold enrichment (to IGG) in the PND 3 group in the third region of the promoter (**Figure 4B**). ANOVA revealed a significant increase in histone acetylation (F_2,5_ = 98.86, p < 0.0001), with PND 56 having four-fold higher levels of H3K9/K14Ac in region three of the promoter compared to PND 3 (p = 0.0002).

### Transcription Factor mRNA and Promoter Binding of Nurr1 and Pitx3 Are Increased at Postnatal day 56 in the rat Midbrain

**Figure 5:**
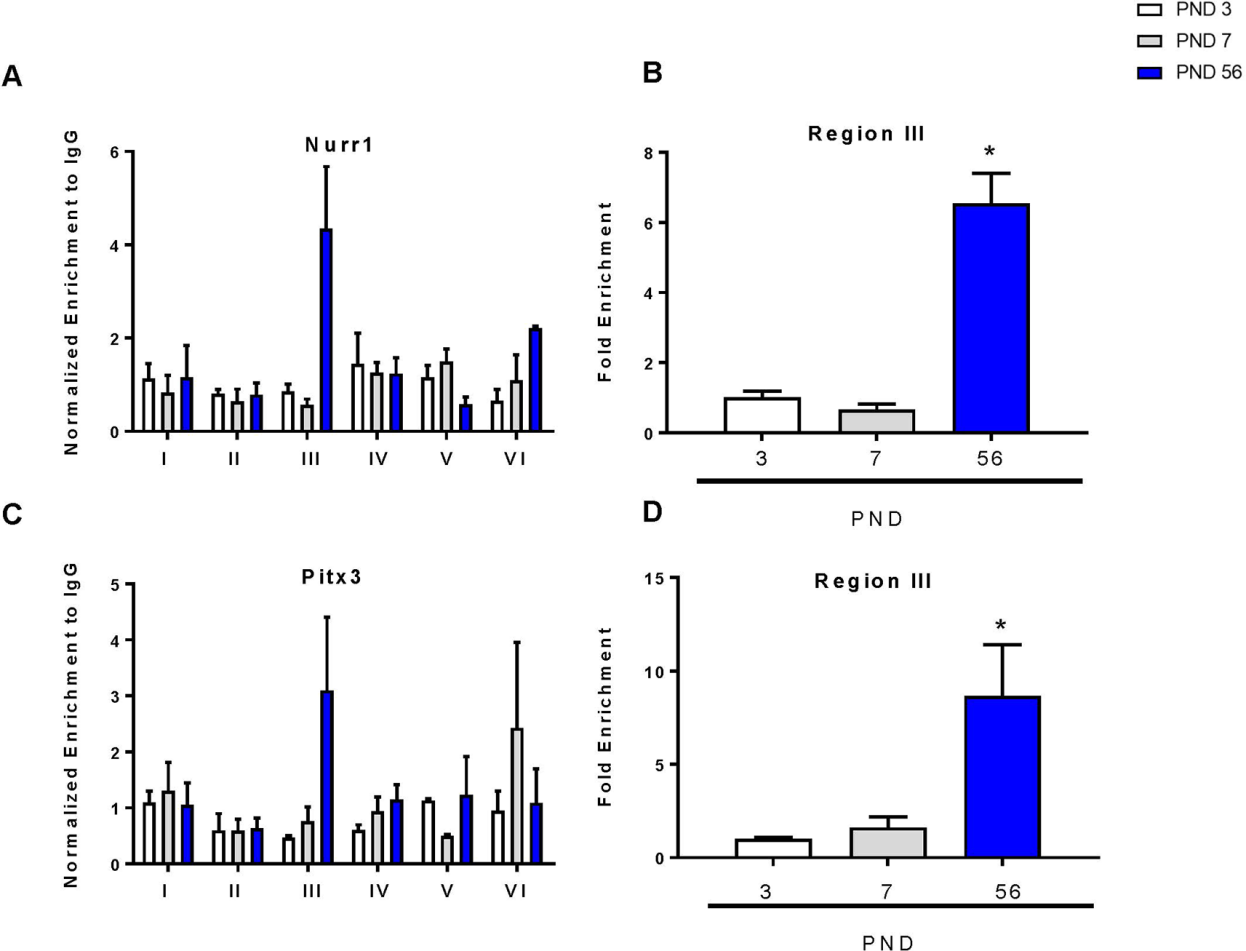
Chromatin immunoprecipitation of Nurr1 and Pitx3 within the DAT promoter. **(A)** Enrichment of Nurr1 across six regions of the DAT promoter normalized to IgG. **(B)** Fold enrichment of Nurr1 relative to PND 3 in the third region of the DAT promoter. **(C)** Enrichment of Pitx3 across six regions of the DAT promoter normalized to IgG. **(D)** Fold enrichment of Pitx3 relative to PND 3 in the third region of the DAT promoter. Data for all graphs are presented as ± SEM; N = 3–4 *p ≤ 0.05 and were analyzed by One-way ANOVA with Dunnett’s *post-hoc* relative to PND 3 for enrichment.

To determine the relative binding of each transcription factor during development, we performed chromatin immunoprecipitation of the DAT promoter with Nurr1 and Pitx3 over 6 regions of the promoter as previously described (He et al., 2011; Green et al., 2017). There were no changes in Nurr1 or Pitx3 binding between PND 3 and 7 (**Figures 5A, C**). For both proteins, region III of the promoter showed the most abundant enrichment compared to the other regions of the promoter. This is consistent with others showing that enrichment of Nurr1 and Pitx3 in the DAT promoter can cooperatively enhance expression (Martinat et al., 2006). ANOVA revealed a significant increase in Nurr1 binding (F_2,7_ = 79.47, p < 0.0001) and in Pitx3 binding (F_2,7_ = 8.13, p = 0.0150) in the third region. These data were graphed to show enrichment relative to PND 3 for both transcription factors. At PND 56 we measured a significant six-fold increase in Nurr1 (p < 0.0001) and an eight-fold increase in Pitx3 (p = 0.0165) (**Figures 5B, D**).

## Discussion

The DAT is primarily expressed in the plasma membrane and smooth endoplasmic reticulum of dendrites in the substantia nigra, as well as in the plasma membrane of axons and axon terminals in the striatum of the rat brain (Nirenberg et al., 1996). It is responsible for the reuptake of dopamine from the synaptic cleft into the presynaptic terminal and therefore involved in a key mechanism in the termination of dopaminergic signaling. Following reuptake, dopamine can re-enter the neuron where it is recycled into synaptic vesicles (Mchugh and Buckley, 2015). As a key regulator of dopaminergic transmission, the normal expression and proper function of the DAT is essential for maintaining dopamine homeostasis. Aberration in its expression or function is linked to different neurodevelopmental disorders. Understanding the mechanisms involved in the developmental regulation of the DAT may aid in identifying novel avenues to correct the abnormal levels presented in disease states.

We measured the relative expression levels of DAT mRNA during various times of postnatal development. A significant increase in DAT mRNA was observed, and the highest surge was measured by PND 56. Other studies report that PND 56 in the rat corresponds to early human adulthood (Sengupta, 2013). Our results differ from previous DAT ontogeny studies, which used *in situ* hybridization and autoradiography techniques. These groups reported maximum expression of DAT at PND 14 (Fujita et al., 1993), PND 21 (Coulter et al., 1996) or PND 28 (Galineau et al., 2004) in contrast to our findings at PND 56. These differences may be explained by older, less sensitive, and less quantitative technologies compared to qPCR. Based on our findings, we selected PND 3, 7, and 56 as low, intermediate, and high expression periods for DAT. These time points were also used in the previous studies. The midbrain dopaminergic neurons project to the striatum where the highest levels of DAT protein are detected. Autoradiography studies using radiolabeled DAT substrates were used to identify regions rich in functional DAT protein (Moll et al., 2000; Galineau et al., 2004). We report significantly higher protein levels of DAT within the striatum at PND 7 and PND 56, with PND 56 showing a maximum for both protein and mRNA levels.

To identify the mechanisms that may be involved in regulating DAT mRNA expression, we explored changes in epigenetic mechanisms in the midbrain. The concept of epigenetics is used to describe both heritable and non-heritable changes in the genome without an alteration to the DNA sequence (Gapp et al., 2014). These mechanisms are involved in regulating gene expression throughout the lifespan, including but not limited to periods of development, tissue differentiation, and in disease (Li, 2002; Jakovcevski and Akbarian, 2012). Of these mechanisms, DNA methylation describes the addition of a methyl chemical group by DNA methyl transferases (DNMTs) to 5-C of cytosines located next to a guanine nucleotide, collectively termed CpG dinucleotide sequences (Meehan et al., 1992). The addition of methyl groups to the promoter region of a gene increases the methylation of that promoter and is generally associated with transcriptional silencing (Meehan et al., 1992; Esteller, 2007). During development, DNA methylation serves to epigenetically program genes and alter expression resulting in defined cell identity (Razin and Riggs, 1980; Teschendorff et al., 2010; Van Montfoort et al., 2012). Post-translational histone modifications work in tandem with DNA methylation to regulate gene expression and chromatin structure. Histone acetylation is largely associated with relaxed chromatin structure and active gene transcription (Haberland et al., 2009b). Histone deacetylases (HDACs) catalyze the removal of acetyl groups from histone tails, which results in the loosening of higher order chromatin and allowing the gene region of interest to become accessible for transcription (Eberharter and Becker, 2002). As we have previously reported in our *in vitro* studies, DNMTs as well as HDACs are involved in the regulation and modulation of DAT expression, therefore we sought to investigate their involvement in the *in vivo* regulation of DAT (Green et al., 2015; Green et al., 2017).

We evaluated the expression of DNMTs (*Dnmt1, Dnmt3a, Dnmt3b*) and class I HDACs (*Hdac1, Hdac2, Hdac3, and Hdac8*) and the class IIa HDAC, *Hdac5* genes in the rat midbrain. We show an age-related decrease in mRNA expression for all three genes examined by PND 56 relative to PND 3. This is consistent with others’ findings demonstrating that DNMT expression typically diminishes in expression following terminal differentiation and maturation of neurons (Goto et al., 1994; Deng and Szyf, 1999; Feng et al., 2005). We evaluated the expression of HDAC mRNA during PND 3, 7, and 56 of rat brain development. HDAC classes are characterized based on a combination of structural differences and location within the cell (Morris and Monteggia, 2013). We chose to evaluate all class I HDACs as they are abundantly expressed in the brain. Furthermore, these HDACs are critically important for development as displayed by lethality in HDAC knockout models during embryonic development or during early postnatal days (Lagger et al., 2002; Bhaskara et al., 2008; Montgomery et al., 2008; Morris and Monteggia, 2013), with the exception of a knockout model of HDAC8 which displays cranial defects, but is otherwise viable (Haberland et al., 2009a). We report no change in expression between *Hdac1* and *Hdac3*, but a decrease in *Hdac2* mRNA by 20% at PND 7 and by 50% at PND 56 relative to PND 3. Conversely, *Hdac8* demonstrated a significant increase in mRNA expression at PND 56. The Class IIa HDAC5 is highly expressed in the brain, and unlike the class I HDACs the knockout models are viable (Renthal et al., 2007). The major behavioral phenotype of this animal model is enhanced cocaine sensitivity, which suggests a relationship between HDAC5 and the dopaminergic system (Renthal et al., 2007). In our studies, we observed an increase in *Hdac5* expression. These data suggest that epigenetic factors may play a role in the ontogenic regulation of DAT.

The dopaminergic transcription factors Nurr1 and Pitx3 are expressed early in midbrain dopaminergic cell differentiation (Smits et al., 2003; Smidt et al., 2004). Their mRNA expression persists within the midbrain throughout life and is essential for proper differentiation and maturation of terminal midbrain dopaminergic neurons. DAT is a target gene of Nurr1 and Pitx3 (Sacchetti et al., 2001; Hwang et al., 2009), therefore the two play a major role in the transcriptional regulation of DAT expression. Mice deficient in Pitx3 have significantly decreased levels of DAT (Hwang et al., 2009), as well as the inability to develop dopaminergic neurons in the substantia nigra (Nunes et al., 2003; Kim et al., 2014). Similarly, mice deficient in Nurr1 also lack midbrain dopaminergic neurons (Jankovic et al., 2005), and deletion of Nurr1 from mature dopaminergic adult neurons increases dopaminergic cell loss, with the neurons of the substantia nigra being more susceptible (Jiang et al., 2005). We examined expression profiles of both Nurr1 and Pitx3 in the rat midbrain and found that Pitx3 mRNA is significantly increased at both PND 7 and PND 56 of these animals. Collectively, these expression profiles suggest that some of these decreases in DNMTs and HDACs, and the increase of transcriptions factors may be participating in the increased expression of DAT, as they decrease at the same timepoint as we measure maximum DAT expression. These data are consistent with our previous findings *in vitro*, which have shown that increases in mRNA of Nurr1 and Pitx3 are also accompanied by an increase in DAT mRNA (Green et al., 2017).

The actions of these epigenetic factors on directly affecting DAT mRNA expression and ultimately protein levels need to be examined at the promoter level. While evaluating changes in gene expression profiles within the midbrain are valuable and provide us with information about the levels of these epigenetic enzymes, they limit the conclusions drawn about the direct epigenetic modifications and transcription factor binding that occurs on the DAT promoter. *In silico* analyses demonstrated potential epigenetic regulation of DAT due to CpG-rich regions within the promoter (Shumay et al., 2010). These studies suggested that the lack of a conserved TATA box within the DAT promoter also allows the potential for gene regulation by histone acetylation (Choi and Kim, 2008). DNA methylation is generally associated with transcriptional silencing. Our pyrosequencing results show an unmethylated DAT promoter across all postnatal days. This promoter region was selected for analysis because of its proximity to the *in vitro* tested binding regions of the dopaminergic transcription factors Nurr1 and Pitx3 (Martinat et al., 2006; Yi et al., 2014). Previous studies indicate reduced affinity of transcription factors to their promoter binding sites due to cytosine methylation (Tate and Bird, 1993; Kim et al., 2003; Perini et al., 2005; Choy et al., 2010). Direct alterations in DAT acetylation were determined by ChIP of the DAT promoter with an acetylated histone mark (H3K9/K14Ac), in order to examine regions of the promoter that may be sites of active transcription (Karmodiya et al., 2012). Our data indicate significant enrichment of H3K9/K14Ac at PND 56 in the third region of the DAT promoter. Both Nurr1 and Pitx3 demonstrated significant binding and enrichment with the same region of the DAT promoter. An unmethylated promoter and increased histone acetylation within the DAT promoter could make transcription factor binding more accessible, resulting in induced DAT expression. Together, these epigenetic modifications along with transcription factor binding may increase DAT mRNA by directly acting on the promoter region.

These data collectively point to epigenetic mechanisms and transcription factors regulating the ontogenic expression of DAT. We report age-dependent differences in DAT gene expression in the developing rat midbrain. Different expression levels of epigenetic modifier genes suggest a diverse role of these genes during midbrain development. Direct assessments of the DAT promoter confirmed previous findings of transcription factor binding. This solidifies the role of Nurr1 and Pitx3 not only in development, but also in adulthood (PND 56). Age-related reductions in DAT promoter methylation correspond to increased gene expression. In the rat midbrain, the regulation of DAT includes the dopaminergic transcription factors Nurr1, Pitx3 and histone acetylation events. In concert these data contribute to a better understanding of mechanisms by which the DAT gene is regulated during development. As DAT is one of the primary modulators of dopamine levels, this understanding may assist in the therapeutic targeting of molecular components, which contribute to the etiology of neurodevelopmental disorders involving dopaminergic dysregulation.

## Data Availability Statement

The datasets generated for this study are available on request to the corresponding author.

## Ethics Statement

The animal study was reviewed and approved by the International Animal Care and Use Committee at Rutgers University that approved all animal handling procedures.

## Author Contributions

AG, LZ, HZ, GG, and JR designed and planned all experiments presented in the manuscript. LZ and AG performed experiments. AE organized and prepared graphical data and performed all statistical analysis. AG, AE, and JR prepared and wrote the manuscript. LZ, HZ, and GG reviewed and edited the manuscript.

## Funding

The authors gratefully acknowledge the support of the NIH grants GM04030, R01ES015991, R01ES021800, P30ES005022, T32ES007148

## Conflict of Interest

The authors declare that the research was conducted in the absence of any commercial or financial relationships that could be construed as a potential conflict of interest.

## References

[B1] Bannon M. J. Michelhaugh S. K. Wang J. Sacchetti P. (2001). The human dopamine transporter gene: gene organization, transcriptional regulation, and potential involvement in neuropsychiatric disorders. Eur. Neuropsychopharmacol. 11, 449–455. 10.1016/S0924-977X(01)00122-5 11704422

[B2] Bhaskara S. Chyla B. J. Amann J. M. Knutson S. K. Cortez D. Sun Z. W. (2008). Deletion of histone deacetylase 3 reveals critical roles in S phase progression and DNA damage control. Mol. Cell 30, 61–72. 10.1016/j.molcel.2008.02.030 18406327 PMC2373760

[B3] Cerruti C. Walther D. M. Kuhar M. J. Uhl G. R. (1993). Dopamine transporter mRNA expression is intense in rat midbrain neurons and modest outside midbrain. Brain Res. Mol. Brain Res. 18, 181–186. 10.1016/0169-328X(93)90187-T 8479287

[B4] Chen N. Reith M. E. (2000). Structure and function of the dopamine transporter. Eur. J. Pharmacol. 405, 329–339. 10.1016/S0014-2999(00)00563-X 11033338

[B5] Choi J. K. Kim Y. J. (2008). Epigenetic regulation and the variability of gene expression. Nat. Genet. 40, 141–147. 10.1038/ng.2007.58 18227874

[B6] Choy M. K. Movassagh M. Goh H. G. Bennett M. R. Down T. A. Foo R. S. (2010). Genome-wide conserved consensus transcription factor binding motifs are hyper-methylated. BMC Genomics 11, 519. 10.1186/1471-2164-11-519 20875111 PMC2997012

[B7] Coulter C. L. Happe H. K. Murrin L. C. (1996). Postnatal development of the dopamine transporter: a quantitative autoradiographic study. Brain Res. Dev. Brain Res. 92, 172–181. 10.1016/0165-3806(96)00004-1 8738124

[B8] Coulter C. L. Happe H. K. Murrin L. C. (1997). Dopamine transporter development in postnatal rat striatum: an autoradiographic study with [3H]WIN 35,428. Brain Res. Dev. Brain Res. 104, 55–62. 10.1016/S0165-3806(97)00135-1 9466707

[B9] Dela Pena I. Gevorkiana R. Shi W. X. (2015). Psychostimulants affect dopamine transmission through both dopamine transporter-dependent and independent mechanisms. Eur. J. Pharmacol. 764, 562–570. 10.1016/j.ejphar.2015.07.044 26209364 PMC4600454

[B10] Deng J. Szyf M. (1999). Downregulation of DNA (cytosine-5-)methyltransferase is a late event in NGF-induced PC12 cell differentiation. Brain Res. Mol. Brain Res. 71, 23–31. 10.1016/S0169-328X(99)00147-3 10407183

[B11] Eberharter A. Becker P. B. (2002). Histone acetylation: a switch between repressive and permissive chromatin. Second in review series on chromatin dynamics. EMBO Rep. 3, 224–229. 10.1093/embo-reports/kvf053 11882541 PMC1084017

[B12] Esteller M. (2007). Cancer epigenomics: DNA methylomes and histone-modification maps. Nat. Rev. Genet. 8, 286–298. 10.1038/nrg2005 17339880

[B13] Falkenburger B. H. Barstow K. L. Mintz I. M. (2001). Dendrodendritic inhibition through reversal of dopamine transport. Science 293, 2465–2470. 10.1126/science.1060645 11577238

[B14] Feng J. Chang H. Li E. Fan G. (2005). Dynamic expression of *de novo* DNA methyltransferases Dnmt3a and Dnmt3b in the central nervous system. J. Neurosci. Res. 79, 734–746. 10.1002/jnr.20404 15672446

[B15] Fujita M. Shimada S. Nishimura T. Uhl G. R. Tohyama M. (1993). Ontogeny of dopamine transporter mRNA expression in the rat brain. Brain Res. Mol. Brain Res. 19, 222–226. 10.1016/0169-328X(93)90031-J 8412565

[B16] Galineau L. Kodas E. Guilloteau D. Vilar M. P. Chalon S. (2004). Ontogeny of the dopamine and serotonin transporters in the rat brain: an autoradiographic study. Neurosci. Lett. 363, 266–271. 10.1016/j.neulet.2004.04.007 15182957

[B17] Gapp K. Woldemichael B. T. Bohacek J. Mansuy I. M. (2014). Epigenetic regulation in neurodevelopment and neurodegenerative diseases. Neuroscience 264, 99–111. 10.1016/j.neuroscience.2012.11.040 23256926

[B18] Giros B. Caron M. G. (1993). Molecular characterization of the dopamine transporter. Trends Pharmacol. Sci. 14, 43–49. 10.1016/0165-6147(93)90029-J 8480373

[B19] Giros B. El Mestikawy S. Bertrand L. Caron M. G. (1991). Cloning and functional characterization of a cocaine-sensitive dopamine transporter. FEBS Lett. 295, 149–154. 10.1016/0014-5793(91)81406-X 1765147

[B20] Goodwin J. S. Larson G. A. Swant J. Sen N. Javitch J. A. Zahniser N. R. (2009). Amphetamine and methamphetamine differentially affect dopamine transporters *in vitro* and *in vivo*. J. Biol. Chem. 284, 2978–2989. 10.1074/jbc.M805298200 19047053 PMC2631950

[B21] Gorentla B. K. Vaughan R. A. (2005). Differential effects of dopamine and psychoactive drugs on dopamine transporter phosphorylation and regulation. Neuropharmacology 49, 759–768. 10.1016/j.neuropharm.2005.08.011 16181646

[B22] Goto K. Numata M. Komura J. I. Ono T. Bestor T. H. Kondo H. (1994). Expression of DNA methyltransferase gene in mature and immature neurons as well as proliferating cells in mice. Differentiation 56, 39–44. 10.1007/s002580050019 8026645

[B23] Green A. L. Hossain M. M. Tee S. C. Zarbl H. Guo G. L. Richardson J. R. (2015). Epigenetic regulation of dopamine transporter mRNA expression in human neuroblastoma cells. Neurochem. Res. 40, 1372–1378. 10.1007/s11064-015-1601-6 25963949 PMC4745645

[B24] Green A. L. Zhan L. Eid A. Zarbl H. Guo G. L. Richardson J. R. (2017). Valproate increases dopamine transporter expression through histone acetylation and enhanced promoter binding of Nurr1. Neuropharmacology 125, 189–196. 10.1016/j.neuropharm.2017.07.020 28743636 PMC5585058

[B25] Haberland M. Mokalled M. H. Montgomery R. L. Olson E. N. (2009a). Epigenetic control of skull morphogenesis by histone deacetylase 8. Genes Dev. 23, 1625–1630. 10.1101/gad.1809209 19605684 PMC2714711

[B26] Haberland M. Montgomery R. L. Olson E. N. (2009b). The many roles of histone deacetylases in development and physiology: implications for disease and therapy. Nat. Rev. Genet. 10, 32–42. 10.1038/nrg2485 19065135 PMC3215088

[B27] He X. B. Yi S. H. Rhee Y. H. Kim H. Han Y. M. Lee S. H. (2011). Prolonged membrane depolarization enhances midbrain dopamine neuron differentiation *via* epigenetic histone modifications. Stem Cells 29, 1861–1873. 10.1002/stem.739 21922608

[B28] Hoebeeck J. Speleman F. Vandesompele J. (2007). Real-time quantitative PCR as an alternative to Southern blot or fluorescence *in situ* hybridization for detection of gene copy number changes. Methods Mol. Biol. 353, 205–226. 10.1385/1-59745-229-7:205 17332643

[B29] Hossain M. M. Richardson J. R. (2011). Mechanism of pyrethroid pesticide-induced apoptosis: role of calpain and the ER stress pathway. Toxicol. Sci. 122, 512–525. 10.1093/toxsci/kfr111 21555338 PMC3155085

[B30] Hwang D. Y. Hong S. Jeong J. W. Choi S. Kim H. Kim J. (2009). Vesicular monoamine transporter 2 and dopamine transporter are molecular targets of Pitx3 in the ventral midbrain dopamine neurons. J. Neurochem. 111, 1202–1212. 10.1111/j.1471-4159.2009.06404.x 19780901 PMC4896488

[B31] Ingram S. L. Prasad B. M. Amara S. G. (2002). Dopamine transporter-mediated conductances increase excitability of midbrain dopamine neurons. Nat. Neurosci. 5, 971–978. 10.1038/nn920 12352983

[B32] Jakovcevski M. Akbarian S. (2012). Epigenetic mechanisms in neurological disease. Nat. Med. 18, 1194–1204. 10.1038/nm.2828 22869198 PMC3596876

[B33] Jankovic J. Chen S. Le W. D. (2005). The role of Nurr1 in the development of dopaminergic neurons and Parkinson’s disease. Prog. Neurobiol. 77, 128–138. 10.1016/j.pneurobio.2005.09.001 16243425

[B34] Jiang C. Wan X. He Y. Pan T. Jankovic J. Le W. (2005). Age-dependent dopaminergic dysfunction in Nurr1 knockout mice. Exp. Neurol. 191, 154–162. 10.1016/j.expneurol.2004.08.035 15589522

[B35] Kahlig K. M. Galli A. (2003). Regulation of dopamine transporter function and plasma membrane expression by dopamine, amphetamine, and cocaine. Eur. J. Pharmacol. 479, 153–158. 10.1016/j.ejphar.2003.08.065 14612146

[B36] Karmodiya K. Krebs A. R. Oulad-Abdelghani M. Kimura H. Tora L. (2012). H3K9 and H3K14 acetylation co-occur at many gene regulatory elements, while H3K14ac marks a subset of inactive inducible promoters in mouse embryonic stem cells. BMC Genomics 13, 424. 10.1186/1471-2164-13-424 22920947 PMC3473242

[B37] Kilty J. E. Lorang D. Amara S. G. (1991). Cloning and expression of a cocaine-sensitive rat dopamine transporter. Science 254, 578–579. 10.1126/science.1948035 1948035

[B38] Kim J. Kollhoff A. Bergmann A. Stubbs L. (2003). Methylation-sensitive binding of transcription factor YY1 to an insulator sequence within the paternally expressed imprinted gene, Peg3. Hum. Mol. Genet. 12, 233–245. 10.1093/hmg/ddg028 12554678

[B39] Kim K. S. Kang Y. M. Kang Y. Park T. S. Park H. Y. Kim Y. J. (2014). Pitx3 deficient mice as a genetic animal model of co-morbid depressive disorder and parkinsonism. Brain Res. 1552, 72–81. 10.1016/j.brainres.2014.01.023 24480473

[B40] Kimmel H. L. Carroll F. I. Kuhar M. J. (2000). Dopamine transporter synthesis and degradation rate in rat striatum and nucleus accumbens using RTI-76. Neuropharmacology 39, 578–585. 10.1016/S0028-3908(99)00160-4 10728879

[B41] Lagger G. O’carroll D. Rembold M. Khier H. Tischler J. Weitzer G. (2002). Essential function of histone deacetylase 1 in proliferation control and CDK inhibitor repression. EMBO J. 21, 2672–2681. 10.1093/emboj/21.11.2672 12032080 PMC126040

[B42] Lauder J. M. Bloom F. E. (1974). Ontogeny of monoamine neurons in the locus coeruleus, Raphe nuclei and substantia nigra of the rat. I. Cell differentiation. J. Comp. Neurol. 155, 469–481. 10.1002/cne.901550407 4847734

[B43] Lee H. S. Bae E. J. Yi S. H. Shim J. W. Jo A. Y. Kang J. S. (2010). Foxa2 and Nurr1 synergistically yield A9 nigral dopamine neurons exhibiting improved differentiation, function, and cell survival. Stem Cells 28, 501–512. 10.1002/stem.294 20049900

[B44] Li E. (2002). Chromatin modification and epigenetic reprogramming in mammalian development. Nat. Rev. Genet. 3, 662–673. 10.1038/nrg887 12209141

[B45] Li J. Dani J. A. Le W. (2009). The role of transcription factor Pitx3 in dopamine neuron development and Parkinson’s disease. Curr. Top. Med. Chem. 9, 855–859. 10.2174/156802609789378236 19754401 PMC2872921

[B46] Lin Z. Canales J. J. Bjorgvinsson T. Thomsen M. Qu H. Liu Q. R. (2011). Monoamine transporters: vulnerable and vital doorkeepers. Prog. Mol. Biol. Transl. Sci. 98, 1–46. 10.1016/B978-0-12-385506-0.00001-6 21199769 PMC3321928

[B47] Livak K. J. Schmittgen T. D. (2001). Analysis of relative gene expression data using real-time quantitative PCR and the 2(-Delta Delta C(T)) Method. Methods 25, 402–408. 10.1006/meth.2001.1262 11846609

[B48] Makkonen I. Kokki H. Kuikka J. Turpeinen U. Riikonen R. (2011). Effects of fluoxetine treatment on striatal dopamine transporter binding and cerebrospinal fluid insulin-like growth factor-1 in children with autism. Neuropediatrics 42, 207–209. 10.1055/s-0031-1291242 22015434

[B49] Martinat C. Bacci J. J. Leete T. Kim J. Vanti W. B. Newman A. H. (2006). Cooperative transcription activation by Nurr1 and Pitx3 induces embryonic stem cell maturation to the midbrain dopamine neuron phenotype. Proc. Natl. Acad. Sci. U.S.A. 103, 2874–2879. 10.1073/pnas.0511153103 16477036 PMC1413837

[B50] Mchugh P. C. Buckley D. A. (2015). The structure and function of the dopamine transporter and its role in CNS diseases. Vitam. Horm. 98, 339–369. 10.1016/bs.vh.2014.12.009 25817874

[B51] Meehan R. Lewis J. Cross S. Nan X. Jeppesen P. Bird A. (1992). Transcriptional repression by methylation of CpG. J. Cell Sci. Suppl. 16, 9–14. 10.1242/jcs.1992.Supplement_16.2 1297654

[B52] Moll G. H. Mehnert C. Wicker M. Bock N. Rothenberger A. Ruther E. (2000). Age-associated changes in the densities of presynaptic monoamine transporters in different regions of the rat brain from early juvenile life to late adulthood. Brain Res. Dev. Brain Res. 119, 251–257. 10.1016/S0165-3806(99)00182-0 10675775

[B53] Montgomery R. L. Potthoff M. J. Haberland M. Qi X. Matsuzaki S. Humphries K. M. (2008). Maintenance of cardiac energy metabolism by histone deacetylase 3 in mice. J. Clin. Invest. 118, 3588–3597. 10.1172/JCI35847 18830415 PMC2556240

[B54] Moore L. D. Le T. Fan G. (2013). DNA methylation and its basic function. Neuropsychopharmacology 38, 23–38. 10.1038/npp.2012.112 22781841 PMC3521964

[B55] Morris M. J. Monteggia L. M. (2013). Unique functional roles for class I and class II histone deacetylases in central nervous system development and function. Int. J. Dev. Neurosci. 31, 370–381. 10.1016/j.ijdevneu.2013.02.005 23466417 PMC3726026

[B56] Nakamura K. Sekine Y. Ouchi Y. Tsujii M. Yoshikawa E. Futatsubashi M. (2010). Brain serotonin and dopamine transporter bindings in adults with high-functioning autism. Arch. Gen. Psychiatry 67, 59–68. 10.1001/archgenpsychiatry.2009.137 20048223

[B57] Nirenberg M. J. Vaughan R. A. Uhl G. R. Kuhar M. J. Pickel V. M. (1996). The dopamine transporter is localized to dendritic and axonal plasma membranes of nigrostriatal dopaminergic neurons. J. Neurosci. 16, 436–447. 10.1523/JNEUROSCI.16-02-00436.1996 8551328 PMC6578661

[B58] Nunes I. Tovmasian L. T. Silva R. M. Burke R. E. Goff S. P. (2003). Pitx3 is required for development of substantia nigra dopaminergic neurons. Proc. Natl. Acad. Sci. U.S.A. 100, 4245–4250. 10.1073/pnas.0230529100 12655058 PMC153078

[B59] Nutt J. G. Carter J. H. Sexton G. J. (2004). The dopamine transporter: importance in Parkinson’s disease. Ann. Neurol. 55, 766–773. 10.1002/ana.20089 15174010

[B60] Perini G. Diolaiti D. Porro A. Della Valle G. (2005). In vivo transcriptional regulation of N-Myc target genes is controlled by E-box methylation. Proc. Natl. Acad. Sci. U.S.A. 102, 12117–12122. 10.1073/pnas.0409097102 16093321 PMC1184034

[B61] Razin A. Riggs A. D. (1980). DNA methylation and gene function. Science 210, 604–610. 10.1126/science.6254144 6254144

[B62] Rego J. C. Syringas M. Leblond B. Costentin J. Bonnet J. J. (1999). Recovery of dopamine neuronal transporter but lack of change of its mRNA in substantia nigra after inactivation by a new irreversible inhibitor characterized *in vitro* and ex vivo in the rat. Br. J. Pharmacol. 128, 51–60. 10.1038/sj.bjp.0702784 10498834 PMC1571617

[B63] Renthal W. Maze I. Krishnan V. Covington H. E. Xiao G. Kumar A. (2007). Histone deacetylase 5 epigenetically controls behavioral adaptations to chronic emotional stimuli. Neuron 56, 517–529. 10.1016/j.neuron.2007.09.032 17988634

[B64] Rodriguez-Traver E. Solis O. Diaz-Guerra E. Ortiz O. Vergano-Vera E. Mendez-Gomez H. R. (2016). Role of Nurr1 in the generation and differentiation of dopaminergic neurons from stem cells. Neurotox. Res. 30, 14–31. 10.1007/s12640-015-9586-0 26678495

[B65] Runyon S. P. Carroll F. I. (2006). Dopamine transporter ligands: recent developments and therapeutic potential. Curr. Top. Med. Chem. 6, 1825–1843. 10.2174/156802606778249775 17017960

[B66] Sacchetti P. Mitchell T. R. Granneman J. G. Bannon M. J. (2001). Nurr1 enhances transcription of the human dopamine transporter gene through a novel mechanism. J. Neurochem. 76, 1565–1572. 10.1046/j.1471-4159.2001.00181.x 11238740

[B67] Salemi S. Baktash P. Rajaei B. Noori M. Amini H. Shamsara M. (2016). Efficient generation of dopaminergic-like neurons by overexpression of Nurr1 and Pitx3 in mouse induced Pluripotent Stem Cells. Neurosci. Lett. 626, 126–134. 10.1016/j.neulet.2016.05.032 27208834

[B68] Schmitt K. C. Reith M. E. (2010). Regulation of the dopamine transporter: aspects relevant to psychostimulant drugs of abuse. Ann. N. Y. Acad. Sci. 1187, 316–340. 10.1111/j.1749-6632.2009.05148.x 20201860

[B69] Sengupta P. (2013). The laboratory rat: relating its age with human’s. Int. J. Prev. Med. 4, 624–630. 23930179 PMC3733029

[B70] Shimada S. Kitayama S. Lin C. L. Patel A. Nanthakumar E. Gregor P. (1991). Cloning and expression of a cocaine-sensitive dopamine transporter complementary DNA. Science 254, 576–578. 10.1126/science.1948034 1948034

[B71] Shimada S. Kitayama S. Walther D. Uhl G. (1992). Dopamine transporter mRNA: dense expression in ventral midbrain neurons. Brain Res. Mol. Brain Res. 13, 359–362. 10.1016/0169-328X(92)90220-6 1352613

[B72] Shumay E. Fowler J. S. Volkow N. D. (2010). Genomic features of the human dopamine transporter gene and its potential epigenetic States: implications for phenotypic diversity. PloS One 5, e11067. 10.1371/journal.pone.0011067 20548783 PMC2883569

[B73] Siciliano C. A. Fordahl S. C. Jones S. R. (2016). Cocaine Self-Administration Produces Long-Lasting Alterations in Dopamine Transporter Responses to Cocaine. J. Neurosci. 36, 7807–7816. 10.1523/JNEUROSCI.4652-15.2016 27466327 PMC4961771

[B74] Smidt M. P. Smits S. M. Burbach J. P. (2004). Homeobox gene Pitx3 and its role in the development of dopamine neurons of the substantia nigra. Cell Tissue Res. 318, 35–43. 10.1007/s00441-004-0943-1 15300495

[B75] Smits S. M. Ponnio T. Conneely O. M. Burbach J. P. Smidt M. P. (2003). Involvement of Nurr1 in specifying the neurotransmitter identity of ventral midbrain dopaminergic neurons. Eur. J. Neurosci. 18, 1731–1738. 10.1046/j.1460-9568.2003.02885.x 14622207

[B76] Spencer T. J. Biederman J. Faraone S. V. Madras B. K. Bonab A. A. Dougherty D. D. (2013). Functional genomics of attention-deficit/hyperactivity disorder (ADHD) risk alleles on dopamine transporter binding in ADHD and healthy control subjects. Biol. Psychiatry 74, 84–89. 10.1016/j.biopsych.2012.11.010 23273726 PMC3700607

[B77] Tarazi F. I. Tomasini E. C. Baldessarini R. J. (1998). Postnatal development of dopamine and serotonin transporters in rat caudate-putamen and nucleus accumbens septi. Neurosci. Lett. 254, 21–24. 10.1016/S0304-3940(98)00644-2 9780082

[B78] Tate P. H. Bird A. P. (1993). Effects of DNA methylation on DNA-binding proteins and gene expression. Curr. Opin. Genet. Dev. 3, 226–231. 10.1016/0959-437X(93)90027-M 8504247

[B79] Teschendorff A. E. Menon U. Gentry-Maharaj A. Ramus S. J. Weisenberger D. J. Shen H. (2010). Age-dependent DNA methylation of genes that are suppressed in stem cells is a hallmark of cancer. Genome Res. 20, 440–446. 10.1101/gr.103606.109 20219944 PMC2847747

[B80] Tison F. Normand E. Bloch B. (1994). Prenatal ontogeny of D2 dopamine receptor and dopamine transporter gene expression in the rat mesencephalon. Neurosci. Lett. 166, 48–50. 10.1016/0304-3940(94)90837-0 7910679

[B81] Usdin T. B. Mezey E. Chen C. Brownstein M. J. Hoffman B. J. (1991). Cloning of the cocaine-sensitive bovine dopamine transporter. Proc. Natl. Acad. Sci. U.S.A. 88, 11168–11171. 10.1073/pnas.88.24.11168 1722321 PMC53095

[B82] Van Montfoort A. P. Hanssen L. L. De Sutter P. Viville S. Geraedts J. P. De Boer P. (2012). Assisted reproduction treatment and epigenetic inheritance. Hum. Reprod. Update 18, 171–197. 10.1093/humupd/dmr047 22267841 PMC3282574

[B83] Vaughan R. A. Foster J. D. (2013). Mechanisms of dopamine transporter regulation in normal and disease states. Trends Pharmacol. Sci. 34, 489–496. 10.1016/j.tips.2013.07.005 23968642 PMC3831354

[B84] Volkow N. D. Wang G. J. Newcorn J. Telang F. Solanto M. V. Fowler J. S. (2007). Depressed dopamine activity in caudate and preliminary evidence of limbic involvement in adults with attention-deficit/hyperactivity disorder. Arch. Gen. Psychiatry 64, 932–940. 10.1001/archpsyc.64.8.932 17679638

[B85] Wang J. Michelhaugh S. K. Bannon M. J. (2007). Valproate robustly increases Sp transcription factor-mediated expression of the dopamine transporter gene within dopamine cells. Eur. J. Neurosci. 25, 1982–1986. 10.1111/j.1460-9568.2007.05460.x 17439486

[B86] Yi S. H. He X. B. Rhee Y. H. Park C. H. Takizawa T. Nakashima K. (2014). Foxa2 acts as a co-activator potentiating expression of the Nurr1-induced DA phenotype *via* epigenetic regulation. Development 141, 761–772. 10.1242/dev.095802 24496614

[B87] Zhu J. Reith M. E. (2008). Role of the dopamine transporter in the action of psychostimulants, nicotine, and other drugs of abuse. CNS Neurol. Disord. Drug Targets 7, 393–409. 10.2174/187152708786927877 19128199 PMC3133725

